# A qualitative analysis of factors influencing physical activity behaviour in women with PCOS: key learning for physical activity interventions and beyond

**DOI:** 10.1093/her/cyae040

**Published:** 2024-12-10

**Authors:** Amie Woodward, Markos Klonizakis, David Broom, Rachel Cholerton, Hilary Piercy

**Affiliations:** Lifestyle, Exercise and Nutrition Improvement (LENI) ResearchGroup, College of Health, Wellbeing and Life Sciences, Sheffield Hallam University, Collegiate Crescent, Sheffield S10 2BP, United Kingdom; Insitute for Health and Care Improvement, York St John University, Lord Mayor's Walk, York YO31 7EX; Lifestyle, Exercise and Nutrition Improvement (LENI) ResearchGroup, College of Health, Wellbeing and Life Sciences, Sheffield Hallam University, Collegiate Crescent, Sheffield S10 2BP, United Kingdom; Centre for Physical Activity, Sport and Exercise Sciences, Health and Wellbeing Research Institute, Coventry University, 20 Whitefriars Street, Coventry CV1 2DS, United Kingdom; Health Research Institute, Sheffield Hallam University, Howard Street, Sheffield S1 1WB; Health Research Institute, Sheffield Hallam University, Howard Street, Sheffield S1 1WB

## Abstract

Physical activity (PA) is recommended in clinical practice guidelines as effective for the management of polycystic ovary syndrome (PCOS). However, adherence to PA interventions is low in this population, and long-term uptake of PA is a challenge. We conducted a feasibility trial of two PA interventions for women with PCOS. This paper reports a qualitative evaluation of the trial in tandem with an evaluation of barriers and facilitators to PA in a sub-group of participants. Eleven participants with PCOS were purposively sampled from the main sample (*n* = 36) and participated in semi-structured interviews. Interviews were audio-recorded and transcribed verbatim. Data were analysed using reflexive thematic analysis. Five themes were developed relating to experiences of the intervention and factors influencing PA behaviour: (1) The Changing Nature of Priorities, (2) The Push and Pull of PCOS Symptoms, (3) Focusing Beyond the Scale, (4) Knowledge as a Foundation for Change and (5) The Balance of Stigma and Social Support. These findings can be used to design PA interventions that consider the interplay between PCOS and PA behaviour to achieve health benefits beyond short-term interventions.

## Introduction

Polycystic ovary syndrome (PCOS) is a complex endocrinopathy affecting 10–13% of reproductive-aged women [1]. It is associated with increased cardiometabolic risk, depression and anxiety disorders and reproductive variability [2, 3].

The international PCOS guidelines [3] recommend physical activity (PA) in the treatment of PCOS. Many PA interventions have been conducted, with a recent systematic review identifying 46 trials using searches up to April 2020 [4]. PA interventions improve a variety of health-related outcomes, including cardiometabolic risk factors, reproductive capacity markers and health-related quality of life in PCOS [5, 6].

However, many intervention studies have small sample sizes, low quality and low adherence [7]. Women with PCOS, particularly those with overweight/obesity and depressive symptoms, have low adherence and high attrition to PA interventions [8]. This may reflect PCOS-specific barriers to engagement in PA, such as lack of confidence and perceived physical limitations [9].

These limitations make it difficult to translate the reported benefits of such interventions into sustained, long-term lifestyle changes that are likely to improve health. Engagement in PA to meet health guidelines in adults is low globally, particularly in women [10]. There is a need for increased participant support for women with PCOS [8] and better design of PA interventions in this population so that health benefits can be achieved beyond short-term interventions.

To improve participation and engagement with PA, interventions should be effective, enjoyable and sustained beyond the intervention [11]. Some PCOS lifestyle interventions integrate behavioural components [12, 13]. Lifestyle interventions usually consist of various components, including diet, therapy and physical activity advice. However, no PA interventions (studies of PA or exercise alone) have utilized behaviour change theories that promote long-term uptake, nor utilized qualitative evaluations. Some studies have explored perceptions, or factors influencing PA, in women with PCOS [14, 15], but these utilized quantitative and/or survey-based methods. In addition, evidence suggests that participating in a PA and lifestyle intervention can reduce perceived barriers to physical activity in PCOS [16]. However, there remains a paucity of literature that utilizes a qualitative approach to evaluate interventions, investigate the factors that affect PA behaviour and the successful (i.e. low attrition, high adherence and a positive experience) implementation of PA interventions in women with PCOS.

We conducted a qualitative evaluation of a PA-focused feasibility randomized controlled trial (RCT) for women with PCOS. The quantitative analysis of feasibility is reported elsewhere [17]. The purpose of this study was to incorporate an exploration of the experiences and factors that influence PA behaviour among women with PCOS participating in a PA trial. This was to elucidate the complex factors that affected the success of our intervention and may affect implementation of future PA interventions in this population.

This research was informed by a pragmatic philosophy; that is, there is no singular interpretation of reality, and the methods selected are goal-oriented and aim to address a real-life issue. This allows flexibility in the methods with the aim to improve future learning and induce change through action.

### Aims and objectives

The aims were as follows:

1. Identify factors that influence PA behaviour in women with PCOS participating in a PA trial.
2. Apply and analyse these factors in tandem with experiences of the intervention to present considerations for better implementation of future interventions.

## Methods

This was a qualitative evaluative study as part of a three-armed feasibility RCT [17]. Women with PCOS (*n* = 36) were randomly allocated to either standard care (control) or one of two 12-wk PA interventions: the exercise group, which was a supervised, aerobic, ramped exercise programme for 2–3 sessions per week, or the lifestyle physical activity group (LPAG), where participants used a smartphone PA-tracking app to measure, improve and report on their daily PA. Data were collected at baseline visit and post-intervention.

### Recruitment and sampling

Purposive sampling was used to achieve variation in trial allocation, age, adherence to the trial and ethnicity to provide a variety of contextual experiences. The sample size was determined to be 12 participants recruited across each study arm. Participants were offered an interview upon completion of the RCT, and attempts were made to contact those lost to follow-up for interview. Eligibility criteria for the overarching trial have been published previously [17].

### Interview schedule

The interview schedule consisted of 12 open-ended questions with probes (see Appendix 1). The initial questions captured demographics, whilst the remainder were informed by literature and explored factors that influence exercise participation in clinical populations. The interview schedule was piloted with one researcher and one lay person, resulting in minor changes to improve coherence.

### Data collection

Semi-structured interviews were chosen as the data collection method. The lead researcher (AW) conducted the supervised exercise sessions, so a second researcher (RC) conducted interviews with these participants to prevent bias arising from relationships built over time. Interviews were 20 to 40 minutes in duration. Nine were conducted via telephone, and two were conducted face-to-face. Interviews were conducted within 2 weeks of trial completion and were audio-recorded using a voice recorder and transcribed verbatim. Interviews were fully anonymized at transcription. Pseudonyms were applied for reporting purposes.

### Data analysis

Data were analysed using reflexive thematic analysis detailed by Braun and Clarke [18]. Reflexive thematic analysis is a data interpretation and analysis tool that can be independent from specific research paradigms and delimited theoretical frameworks [19]. It was considered appropriate because the interpretation of the data was not driven by a pre-defined theoretical framework. The stages of the process are (1) familiarization of the data, (2) generation of codes, (3) combining of codes into themes, (4) reviewing themes, (5) determining the significance of themes and (6) reporting of findings [18].

Data were coded by one researcher (AW), and the concept of ‘critical friends’ was used; this involves gathering feedback and using team members as sounding boards to stimulate exploration and challenge interpretations rather than aiming for consensus [20].

### Quality considerations

Credibility was established using deviant case analysis and the provision of extracts from the data source to demonstrate the data constituting the findings. Transferability was established using purposive sampling for variation and the presentation of demographic data for the reader to consider in relation to transferability of the findings. Dependability was established by demonstrating transparency and clear reporting so that other researchers may follow the process. Member-checking was not conducted due to practical and logistical issues, such as dealing with disagreement, recall issues and whether or not the participant has indeed read the text and not simply deferred to perceived authority [20].

### Ethical considerations

Health Research Authority approval and Research Ethics Committee favourable opinion was granted by the North West—Greater Manchester East REC (18/NW/0454).

## Results

Eleven interviews were conducted: five from the exercise group, four from the LPAG and two from the control group. Although two participants from the control group indicated willingness, they were not available for interview.

Adherence to the exercise program ranged from 18% to 96% (defined as attendance at sessions). Adherence in the LPAG was 100% (defined as measuring and reporting PA data as directed) [17]. Demographic information can be seen in Table I.

**Table I.** Demographic and adherence data

Pseudonym	Age (Years)	Ethnicity	Highest Education	Income Bracket^a^	Allocation	Adherence
Carrie	19	British Pakistani	Undergraduate	£21-30k	A	75%
Corin	19	British Pakistani	Undergraduate	Under £20k	A	18%
Debbie	31	British Asian	Doctorate	£41k+	A	96%
Frances	49	White Other	Post-graduate	£31-40k	A	78%
Janet	26	White British	Undergraduate	£41k+	A	82%
Courtney	35	White British	Doctorate	£41k+	B	100%
Kathleen	29	White British	Undergraduate	£21-30k	B	100%
Tali	34	White British	Undergraduate	£31-40k	B	100%
Shirley	30	White British	Undergraduate	£41k+	B	100%
Dolores	32	White British	Post-graduate	£31-40k	C	n/a
Marissa	34	White British	Level 3	£31-40k	C	n/a

a Annual household income.

Five themes were identified: (1) The Changing Nature of Priorities, (2) The Push and Pull of PCOS Symptoms, (3) Focusing Beyond the Scale, (4) Knowledge as a Foundation for Change and (5) The Balance of Stigma and Social Support.

### The changing nature of priorities

Work and family responsibilities were indicated as barriers to PA, as expected for young to middle-aged women with families, sometimes children and jobs. However, perceptions of ‘enough time’, and when was a good time to exercise varied—some felt that a ‘9–5ʹ job was too constricting with little time or energy to exercise after work, while others who had worked nights before switching to 9–5 felt that they had more time to participate in things like evening classes:

*I feel like I’ve probably got more time to fit in something regularly on an evening cause I work more regular hours (Tali)*.

This indicates that there is no ‘ideal’ working pattern that optimizes PA engagement because participants had differing perceptions on how PA could fit in around work and different expectations about when a time is convenient to exercise based on their responsibilities.

Furthermore, some participants discussed making excuses not to engage in PA despite being presented with opportunities, such as an invitation from friends:

*They’re in a WhatsApp group as well, and they invite me…but I make excuses [laughs] (Dolores)*.

The findings suggest that rather than lack of time, there is a more complex underlying issue of prioritization of PA. Some participants highlighted that they made time, while others indicated that in a busy life, PA fell down the list of priorities:

*when I were younger, I loved … keeping fit … and then it has just sort of fell by wayside cause I’ve got older and you’ve got a house and you’ve got things to do and you’ve got a job and … it just becomes sort of less of a … priority doesn’t it (Tali)*.

The varying prioritization was reflected in the experiences of those who completed the PA interventions. Debbie explained: *‘I prioritised it because it was important’*. The adherence figures showed varying levels of participation despite participants citing similar time commitments, and this supports the idea that prioritization may have been an influential factor.

The key concept of this theme is that priorities differ and evolve, and it is important to consider periodic reassessment and realignment of the intervention with participants’ shifting priorities to ensure continued engagement.

### The push and pull of PCOS symptoms

A key theme was the propensity for PCOS symptoms to act as both facilitator and barrier to PA. Weight gain motivated participants to engage in PA. This was related more to appearance and self-image than health outcomes:

*Normally it’s whenever I got very upset about my body and… you just think, oh that’s it, I’m gonna do some exercise (Carrie)*.

This shows how exercise or PA was used to dispel negative feelings about the body. However, negative feelings and shame also played a role as a deterrent:

*[Be]cause I’d been big since I was…14 to 15, a major thing that always stopped me doing anything about it was shame (Kathleen)*.

Those participants in the PA interventions who had perceived changes in weight had improved self-image. Similarly, unsuccessful attempts to lose weight decreased motivation:

*You don’t enjoy it and you don’t see the benefit [of weight loss] so then you don’t wanna do it again (Marissa)*.

Weight may therefore be a negative reinforcer of PA initially, but associated poor self-image and weight loss frustration cause avoidance, and this may be a barrier to long-term, sustained PA. This cycle is repeated when participants felt particularly negative about their bodies.

Mood perpetuated a similar cycle of motivation and avoidance. Participants acknowledged that their low mood meant they did not feel like exercising, whilst simultaneously knowing that PA could improve their mood if they engaged. When they did not exercise, this worsened their low mood, developing into a ‘vicious cycle’ (Shirley). This was exacerbated by fatigue, which prevented PA and subsequently fed into feelings of low mood. Conversely, when participants did exercise through the LPAG, they noted that they felt energized and more motivated:

*I always felt a bit more energized the day after I’d gone and done my swimming… so I felt a bit more “let’s go for it” (Kathleen)*.

The central concept of this theme is that participants’ relationship to their PCOS symptoms (and bodies) had a complex and contrasting impact on PA behaviour. It suggests that relying on weight-related outcomes would not be a successful strategy for long-term PA engagement.

### Focusing beyond the scale

In keeping with the previous theme, when participants centred the positive effects of PA beyond weight, they were more likely to engage in PA. Taking part in the PA intervention(s) served as a reminder of ‘how good you do feel when you move more’ (Courtney). Similarly, others indicated that seeing benefits from regular PA increased their inclination to keep going after the trial. This healthier attitude was about enjoyment and being mindful of health rather than weight or image. Previously, participants engaged in PA due to negative self-image or ‘not wanting to be fat’. After the trial, there was a shift from using exercise to prevent something ‘negative’ to using it to produce something positive:

*At first, I were purely doing it for, like, aesthetic purposes, whereas now it is more for my health… what’s going on inside rather than the outside (Janet)*.

Participants across both interventions reported increased well-being and confidence. These effects did not seem to be influenced by weight changes:

*It was nice to just kind of think, oh well at least I feel better even if I wasn’t noticing any physical changes like weight wise or size wise (Kathleen)*.

This has important implication for PCOS where weight loss is often difficult to achieve and suggests that weight loss need not be the primary objective of PA to produce positive outcomes.

The key concept of this theme is that when exercise was experienced as a positive force irrespective of visible changes, participants became motivated to engage or continue to engage in PA. It highlights the need to shift the focus from weight-centric outcomes to holistic well-being in PCOS-related PA interventions.

### Knowledge as the foundation for change

Participants expressed frustration at the lack of guidance around effective PA that would address their physical and emotional symptoms. When they sought advice, mainly online, many felt that information was inconsistent and conflicting, and they would benefit from ‘stuff tailored for people like myself [with PCOS]’ (Janet).

Those seeking professional advice felt that fitness professionals and instructors lacked empathy and the knowledge of PCOS as a metabolic condition that comes with challenges surrounding weight loss.

*The only information that I would be given was: you need to lose weight … its not very, realistic because with polycystic ovaries it is hard to lose weight (Dolores)*.

The participants indicated a need to access information, advice and guidelines that takes into consideration the pressures, demands and challenges of PCOS:

*Advice and education is so important … what it is that you’re doing and why that’s beneficial and helpful, because it changes your mindset and motivates you to actually do it (Corin)*.

For those that did know the benefits of PA on physiological processes, such as insulin resistance, this knowledge became part of the motivation to undertake PA:

*Probably is my biggest reason, is like just trying to keep my … insulin levels … you know in … kind of a healthy range so I don’t get all the stuff [diabetes](Frances)*.

The central concept of this theme is that providing participants with the knowledge of how and why PA benefits PCOS symptoms (as well as the limitations) influences their motivation, and this underscores the potential impact of educational components in PA interventions.

### The balance of stigma and social support

The participants expressed a need for social support to increase motivation to engage in PA. Examples included team sport, one-on-one with a friend or family member, and competitions between small groups of friends. Many participants felt that socializing positively influenced PA participation because it was more fun and reduced perceived effort:

*I could always do more if I went with a friend … I’d be sort of like chatting and you don’t sort of notice (Tali)*.

One participant in the LPAG took a friend along each time she exercised and said:

*I think their [friend] support was pretty instrumental in making me go and do stuff (Shirley)*.

However, the desire for social support was threaded through with fears of shame or stigma about their condition or their bodies. One participant described the thought of big group classes as ‘emotionally horrifying’ (Kathleen) and preferred options with less exposure:

*Swimming was nicer cause literally mostly all they could see is my head (Kathleen)*.

Another wished for ‘private gym rooms’ (Corin). These findings do not suggest that the participants would prefer to exercise alone; in fact, many participants had trusted people that served to increase motivation. Rather, it was a need for social support that minimized exposure to the public. The exercise sessions as part of the present intervention were held in a private, ‘research’ gym, one-to-one with the researcher. Participants found this environment to be ‘safer’ than a traditional gym:

*I don’t feel as scared, because it’s not like a normal gym, where there’s just lots of people, and I’ve always felt quite self-conscious (Carrie)*.

However, the desire for social connectivity and commonality from those in the exercise group remained:

*There was never a time when I was like interacting with another participant so you know … it was kind of quite an isolating experience (Frances)*.

The central concept of this theme is the need for social support while preserving the dignity of participants and reducing shame or embarrassment. Interventions that exacerbate shame or embarrassment risk alienating participants and activating a negative spiral that leads to less participation in PA (as in the ‘Push and Pull of PCOS Symptoms’).

## Discussion

PA has many benefits for PCOS, but the literature indicates small sample sizes and poor adherence in PA interventions [21]. This limits the confidence in the findings and represents missed opportunities to support behaviour change. This qualitative study provides detailed insight into participants’ perceived barriers and facilitators to PA and interprets them in line with experiences of participating in the present intervention. Five themes were identified that reflect the individual, physical and social factors that influence participation and engagement in PA. The findings, which are grounded in the experience of the participants, provide important considerations for future design of PA interventions for PCOS to improve adherence, experience and sustained PA behaviour.

### Making physical activity a priority

Participants largely cited time as a barrier to PA. Lack of time and motivation are frequently reported barriers to PA, particularly for women [22]. In line with similar interventions, our exercise intervention had a low adherence of 53% [8]. During the exercise intervention, participants continued to miss sessions despite flexible, advance scheduling. This suggests that whilst lack of time is the perceived external barrier, the actual barrier may be internal, such as the assigned priority to PA [23].

Participant responses supported the idea that prioritization of PA was a barrier. Participants commented that exercise and PA ‘fell by the wayside’ in relation to other priorities and that they ‘made excuses’ despite opportunities. Conversely, some participants in the exercise intervention commented that although it was sometimes difficult to fit in the sessions, they made this a priority because it was ‘important’. These differing priorities may have been due to the age of participants, which ranged from 19 to 49 years. Participants’ priorities are likely to be different based on their life experience and responsibilities (such as work, children) and subsequent support systems. Indeed, older adults are less likely to be physically active than their younger counterparts, and their barriers may be distinct [24].

One finding was that priorities evolved over time, and though PA had been important at various time points, this could change as lifestyles shifted. To align the intervention with the priorities of the participants, interventions could be designed with behaviour change techniques threaded throughout. Some efficacious behaviour change techniques in PA interventions include providing feedback on performance, self-monitoring and goal setting [25]. This practice, with frequent re-evaluation, could help participants set achievable and realistic goals that fit in with competing priorities.

Interventions would benefit from theoretically informed, embedded behaviour change theories (BCTs) or tools such as the Behaviour Change Wheel (BCW). The BCW helps researchers to understand and identify the capability, opportunity and motivation sources of behaviour [26]. The BCW recognizes that sustained and robust behaviour change is affected by a variety of influences and helps researchers to develop interventions that affect long-term behaviour change [26]. Psychological support may also be appropriate alongside BCTs to address the emotional dimensions of PCOS that affect both motivation and avoidance of PA [12].

### Shame and the social environment

Participants desired and were motivated by social connectivity through PA, whether with trusted individuals or other women with PCOS. This meant some participants found the one-to-one (with the researcher) exercise sessions in the intervention to be isolating. However, participants were also vehemently opposed to exercising in big groups or with the public at large. This was due to shame around one’s body. Shame has been documented previously in women with perceived overweight, and a systematic review of studies investigating the link between shame and PA levels found that weight stigma (and the internalization of it) was linked to reduced levels of PA and worse health outcomes [27].

Conversely, research around community-based physical activity events, such as Parkrun, has found that social factors increased enjoyment and perceptions of energy [28]. This research highlights the link between social reward, such as feelings of community and belonging, and exercise-related behaviour that can impact health outcomes [28]. Social support can have a substantial impact on health and induce behaviour change, with research indicating that individuals with low social support have a higher risk of mortality compared with those with stronger social support [29]. The addition of social connectivity into the research design may provide physical and mental health benefits in addition to the PA itself and may influence the development of social support networks that could positively influence the long-term impact of the intervention. Organizing small group sessions could facilitate relationships, enable information sharing and reduce feelings of isolation. This mutual support may make the experience more meaningful, enjoyable and improve adherence rates.

### Knowledge and empowerment

Participants indicated a lack of knowledge around effective PA for their goals (such as weight loss and mitigating PCOS symptoms). This led to frustration and was a barrier to PA. Previous research has found that women with PCOS may have a perception that PA and diet would not be helpful, particularly to lose weight [30]. Although women with PCOS are motivated to make changes, the lack of clear guidance and knowledge can be a hindrance [31, 32].

PA interventions may benefit from incorporating a PCOS-specific educational component that focuses on the successes of previous PA interventions [31, 32]. It could outline the benefits of PA on mental and emotional health in PCOS. This component alongside practical PA sessions may lead to increased empowerment through participation, knowledge, skill and a facilitating environment, as per the World Health Organisation definition of empowerment [33].

Empowerment leads to increased self-management of chronic conditions, resulting in improved clinical outcomes and the development of effective self-care behaviours [34]. This could be enhanced by addressing the psychological aspects of PCOS by signposting or incorporating behavioural treatment or resources [7]. This is recommended by the international PCOS guidelines to optimize weight management, health and emotional wellbeing [3]. For participants to benefit further from PA interventions, researchers should include mental and emotional wellbeing as primary outcomes to ensure interventions are not just designed with weight-loss in mind, as well as including non-weight-related markers of success. These could include improvements in fitness or self-efficacy and may be used with behaviour change techniques such as feedback, self-monitoring and goal setting.

### Strengths and limitations

The main strength and novelty of this study is the use of a qualitative method to clarify the social and personal contexts that influence the success of PA interventions in PCOS. This addresses the current issue of PA interventions in this population of low quality and/or adherence.

A limitation is that the recruitment approach may have discouraged participants with only negative views or experiences of the intervention from participating. This was mitigated to an extent by having an external qualitative researcher conduct interviews with those in the exercise arm.

### Conclusions

This study explores the interplay between PCOS and PA behaviour and uses this in tandem with participants’ experiences of the intervention(s) to present considerations for future research design. Suggestions for improving both the current intervention (and for other such interventions) include embedding BCT(s) to ensure that effective behaviour change techniques and intervention functions are incorporated at the outset. This may lead to increased priority of PA, which is relevant for adherence and the long-term uptake of PA behaviour. Educational and psychological support components could improve empowerment and self-management of PCOS through providing important knowledge, opportunity and promoting emotional well-being. Finally incorporating social connectivity and support in a way that does not exacerbate shame and negative self-image could provide additional benefits to participants during the intervention and beyond.

### Practice implications

The findings may increase the efficacy of PA interventions in PCOS that encourage long-term adherence and promote sustained behaviour change. Long-term participation in regular PA may reduce associated disease risk. Provision of credible educational and psychological support could improve patient self-management, clarity and expectations of intervention.

## Appendix 1. Interview Guide

- Demographics—confirm age, ethnicity (self-identify), highest education level and annual household income (if participant is comfortable answering).

1. Which study arm did you take part in? (closed question—exercise or lifestyle physical activity, or control).
2. Did you complete the study? Did you find it difficult or easy to complete? Why?
3. What did you like (enjoy/find positive) and dislike about the study? How did you overcome any challenges?
4. Did you notice any changes in yourself (probe physical/mental/emotional) throughout or after taking part in the study? How quickly? Positive/Negative changes?
5. Did any of these changes meet/not meet your expectations of what would happen during the study? Why?
6. Which aspect(s) of PCOS has the biggest effect on your life?
7. Did you feel that the intervention had any effect on this aspect(s)?
8. What factors do you feel influenced how regularly you exercised before joining the study?
9. Are you likely to take up regular exercise because of the study? Probe Attitude to exercise?
10. If not, why not?
11. If yes, what kind? How? What kind of support is needed?
12. Would you take part in future research on PCOS and exercise? 
13. If you could take part in an exercise intervention for another study, what factors would you like to be considered? Probe—Location, type of exercise, frequency
